# Are group-based parenting programmes in Sweden a cost-effective way of reducing early child behaviour problems?

**DOI:** 10.1186/1472-6963-14-S2-P107

**Published:** 2014-07-07

**Authors:** Filipa Sampaio, Inna Feldman

**Affiliations:** 1 Department of Women’s and Children’s Health, Uppsala University, 751 25 Uppsala, Sweden

## Background

Child conduct problems increase the risk of costly negative outcomes later in life [1,2]. Parenting programmes are effective in reducing child conduct problems but only few cost-effectiveness studies are published [3]. To our knowledge, there are no cost-effectiveness analyses comparing several parenting programmes in a randomised control trial (RCT).

## Materials and methods

A cost-effectiveness analysis of four programmes, Komet, Connect, the Incredible Years, Cope, and a self-guided book on parenting strategies compared to a waitlist control, was conducted at 4-months post-test, from a payer’s perspective, based on a RCT. The study samples consisted of 961 parents of 3-12 year-old children with conduct problems, including 862 who started a programme or reading a self-guided book, and 159 in the waitlist control. Conduct problems were measured by the Eyberg child behaviour inventory (ECBI). The outcome measures were the incremental cost per one point reduction in the ECBI intensity scale, and incremental cost per one averted clinical case of conduct problems.

## Results

Average intervention cost per child ranged between 120 SEK (£8,91) for the book - 12035 SEK (£893,87) for the Incredible Years. The book and Komet were cost-effective in the reduction of ECBI mean intensity scores with an ICER of 13 SEK (£0,97) and 772 SEK (£57,34) per one ECBI point reduction. Cope was cost-effective targeting the number of averted cases of conduct problems, with an ICER below zero per case averted. Cope also yielded the lowest average cost per averted case, 16322 SEK (£1212,28). Sub-group analyses showed that program completion led to greater cost-effectiveness.

## Conclusions

Different programmes were cost-effective depending on the outcome. The book and Komet were cost-effective in improving child behaviour on a group level, whereas Cope was cost-effective in reducing clinical cases of conduct problems. Selection of the most appropriate programme or combination of programmes should be determined by the aim of the intervention, budget constraints and decision-makers willingness-to-pay.

## References

[B1] Scott S Financial cost of social exclusion: follow up study of antisocial children into adulthood BMJ 2001 323 191 11473907 10.1136/bmj.323.7306.191 PMC35269

[B2] Fergusson DM Horwood LJ Ridde EM Show me the child at seven: the consequences of conduct problems in childhood for psychosocial functioning in adulthood J Child Psychol Psychiatry 2005 14 837 849 10.1111/j.1469-7610.2004.00387.x 16033632

[B3] Dretzke J The effectiveness and cost-effectiveness of parent training/education programmes for the treatment of conduct disorder, including oppositional defiant disorder, in children Health Technol Assess 2005 14 iii ix-x, 1-233 16336845 10.3310/hta9500

